# Surgical Correction and Oral Health-Related Quality of Life (OHRQoL) Evaluation of Non-syndromic Congenital Double Lip: A Case Report and Literature Review

**DOI:** 10.7759/cureus.53446

**Published:** 2024-02-02

**Authors:** Bhawana Oriya, Farrukh Faraz, Arundeep K Lamba, Shruti Tandon, Sachin Dhingra

**Affiliations:** 1 Periodontics, Maulana Azad Institute of Dental Sciences, New Delhi, IND

**Keywords:** ohrqol, hyperplastic tissue, double lip, congenital, non-syndromic, aesthetic

## Abstract

Congenital double lip is a rare clinical finding that is more common in the upper lip, but the lower lip can also be involved in a few cases. It has no gender predilection, and its incidence rate is yet unknown. It can be classified into two types: congenital or acquired. Congenital type results from the persistence of the sulcus between the pars glabrosa and the pars villosa, while the acquired type occurs secondary to trauma and oral habits such as lip sucking and lip biting. It can be syndromic or non-syndromic based on the other systemic involvement. In the present case, a 24-year-old male patient presented to the outpatient department (OPD) with the chief complaint of an excessive tissue fold visible in relation to his upper lip while smiling and talking, which posed an aesthetic concern to the patient and decreased his self-esteem. The oral health-related quality of life (OHRQoL) was taken before and after the treatment to assess patient satisfaction and improvement in quality of life after surgery. The patient was diagnosed with a non-syndromic congenital double lip based on clinical history and oral examination. The surgical removal of excessive lip tissue was done, and the lesion healed completely with no recurrence up to a six-month follow-up. This case report illustrates the uncomplicated surgical treatment for congenital double lips and improves the patient's aesthetic.

## Introduction

A double lip is defined as an excessive fold of lip tissue present at the transition from the oral mucosa to lip vermilion [1]. It is an unusual abnormality also called "macrocheilia" or hamartoma, consisting of a fold of excess or redundant tissue on the mucosal side of the lip [2]. The double lip is usually not evident at rest; however, it is visible when the lips are stretched, such as when smiling and laughing [3,4]. When the upper lip is strained, the tissue frequently projects beyond the vermilion of the lip and takes the shape of a classic "cupid's bow." Double lips can be classified into two types: acquired or congenital. The acquired type may be secondary to trauma or oral habits such as lip biting and lip sucking between diastema, or it can occur due to ill-fitting dentures; however, the congenital double lip is a developmental anomaly [3-5]. It can also be divided into two types: syndromic and non-syndromic. The syndromic type is associated with Ascher syndrome, which is a triad of double lip, blepharochalasis, and nontoxic thyroid goitre [6]. The non-syndromic type of double lip can develop without any underlying condition. It is mostly associated with the upper lip, although the lower lip can also be involved, but in very rare instances, both lips are involved [7]. According to Palma and Taub in 2009, the double lip is more common in males (ratio of 7:1) [8], while most of the authors have not specified any gender, familial, or racial predilection [4,7,9]. Although it may create functional problems, these malformations mostly cause great emotional stress to the affected individual due to the disfigured lip during smiling. Many psychometric tools for evaluating oral health-related quality of life (OHRQoL) have been developed during the last few decades. Assessing OHRQoL in children, adults, and dentate elderly individuals is frequently done with the Oral Health Impact Profile (OHIP) questionnaire [10]. OHRQoL is the subjective experience of symptoms connected to oral problems that affect one's well-being. Management of the double lip is important to correct psychological problems due to aesthetics, speech, mastication, or the ability to wear a prosthesis. We aimed to address the patient's concern about his appearance and provide treatment based on his expectations.

## Case presentation

**Figure 1:**
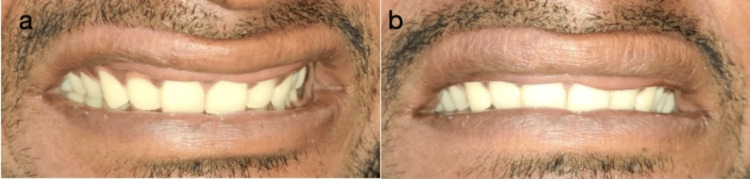
(a-b) Preoperative clinical view and preoperative frontal view showing extra mucosal tissue on accentuation of the upper lip.

A 24-year-old male patient reported to the outpatient department (OPD) of the Department of Periodontics in December 2022 with a chief complaint of excessive tissue fold visible on the upper lip while Tablesmiling and speaking. It was unaesthetic to the patient, which lowered his self-esteem due to continued mocking by colleagues. The patient had been aware of the condition since childhood, and there was no associated syndrome. Family history was non-contributory. On thorough clinical examination, there was an extra bulk of tissue on the mucosal aspect of the upper lip (Figure 1a, 1b).

There was no drooping of the eyelid (blepharochalasis) and no thyroid swelling present. When the patient kept his lips apart while smiling and stretching, it appeared to have a cupid's bow shape. Intraorally, the overlying mucosa was normal in appearance. On palpation, the tissue was soft, mobile, and painless. A provisional diagnosis of non-syndromic congenital double lip was made.

**Figure 2:**
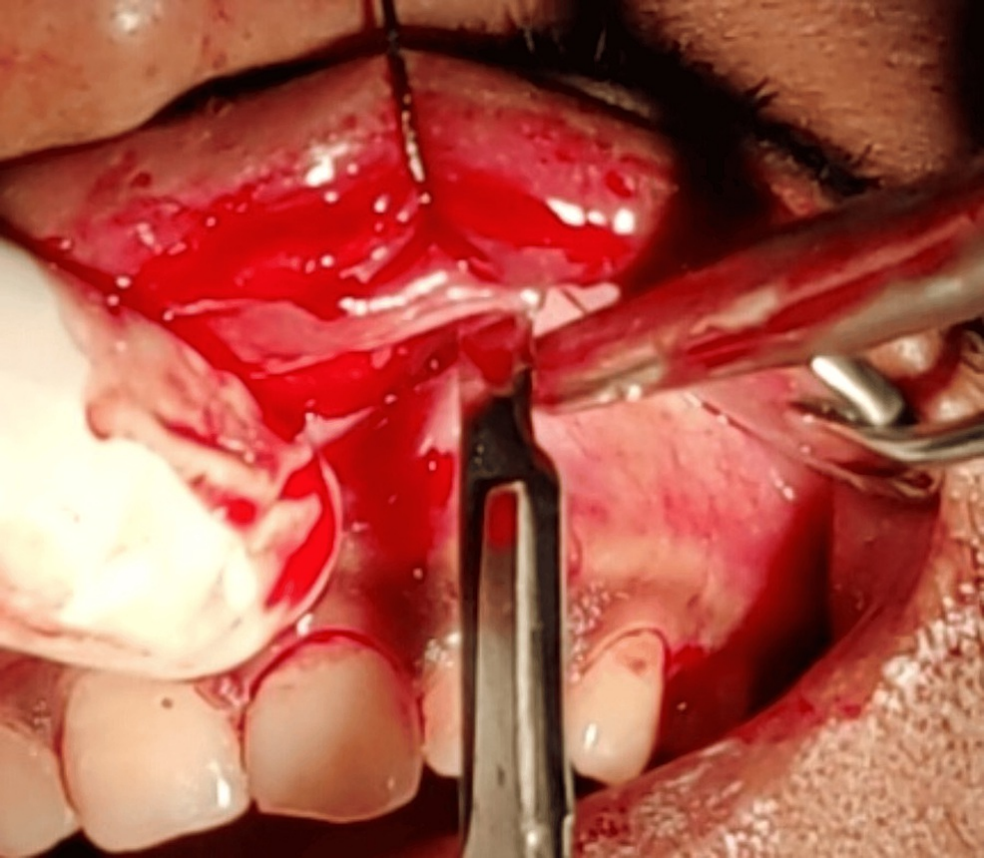
Intraoperative view showing the incision and removal of the excessive lip tissue.

Phase I therapy was done, and the treatment plan was explained to the patient. Written informed consent was obtained before surgical therapy. A bilateral infraorbital nerve block was administered using 2% lignocaine local anaesthesia and adrenaline (1:80,000). An elliptical-shaped marking was made around the extra bulk of tissue using a methylene blue pencil. Two securing sutures were given at both ends of the marking to prevent extra lip tissue loss. The incisions were made along the markings (Figure 2a, 2b).

**Figure 3:**
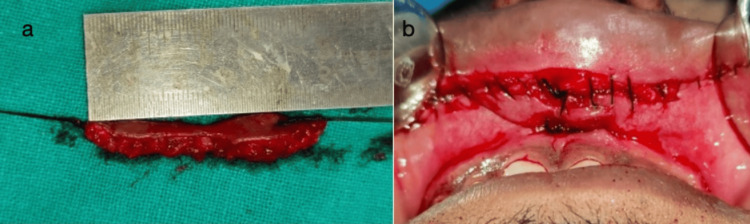
Intraoperative photos showing (a) the removed excessive mucosal tissue and (b) the approximation of tissue with silk sutures after the removal of the excess tissue.

The extra lip tissue was removed. The specimen was sent for histological examination (Figure 3a). Primary closure was achieved with a 3-0 continuous silk suture (Figure 3b).

**Figure 4:**
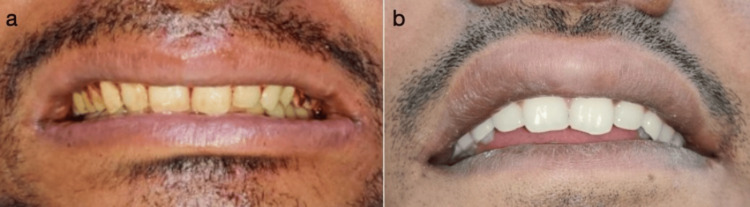
(a) Postoperative photograph of the patient immediately after surgery. (b) Postoperative photograph of the patient after two weeks.

A compression dressing was given over the upper lip to prevent excessive swelling of the lip during the healing phase. For the first four weeks, the patient reported some "tightness" in the lip that resolved with time (Figure 4a, 4b).

The present case report also determined the patient's OHRQoL and self-rated oral health, as well as the effect of the double lip on the patient's psychological state before and after treatment based on the appropriate questionnaire (Table 1 and Table 2).

**Table 1:** Preoperative OHRQoL filled by the patient. OHRQoL: oral health-related quality of life

Domain	Items	Never (0)	Hardly ever (1)	Occasionally (2)	Fairly often (3)	Very often (4)
Functional limitations	Difficulty in pronouncing		+			
	Difficulty in eating			+		
Physical pain	Aching mouth			+		
	Lip biting				+	
Psychological problems	Self-conscious to appearance			+		
	Feeling anxiety				+	
	Feeling depressed					+
Social disability	Lower self-confidence			+		
	Difficulty in doing the usual job				+	
	Avoid going out					+
Handicap	The feeling of life unsatisfaction				+	
	Unable to function			+		

**Table 2:** Postoperative OHRQoL filled by the patient. OHRQoL: oral health-related quality of life

Domain	Items	Never (0)	Hardly ever (1)	Occasionally (2)	Fairly often (3)	Very often (4)
Functional limitations	Difficulty in pronouncing	+				
	Difficulty in eating	+				
Physical pain	Aching mouth	+				
	Lip biting		+			
Psychological problems	Self-conscious to appearance		+			
	Feeling anxiety	+				
	Feeling depressed	+				
Social disability	Lower self-confidence	+				
	Difficulty in doing the usual job	+				
	Avoid going out	+				
Handicap	The feeling of life unsatisfaction		+			
	Unable to function		+			

**Figure 5:**
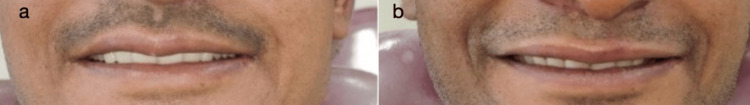
(a) Postoperative photograph of the patient, one month postoperatively. (b) Postoperative photograph of the patient, six months postoperatively.

One-month and six-month follow-ups were done to evaluate the healing of the lip and aesthetics (Figure 5a, 5b).

**Figure 6:**
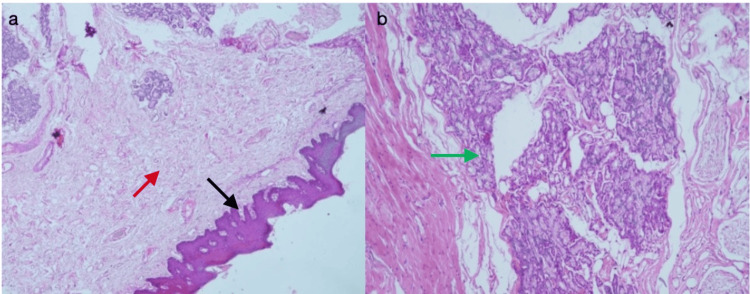
Photomicrographs of hematoxylin and eosin staining. (a) Photomicrograph at low magnification (10×) exhibiting normal mucosal tissue. (b) Photomicrograph at 40× magnification showing normal mucous acini with striated and excretory ducts.

On microscopic examination, the section showed parakeratinized stratified squamous epithelium with long and narrow rete ridges. The underlying connective tissue was moderately to densely collagenous, comprising haphazardly arranged collagen fibres and spindle-shaped fibroblasts. Variable-sized blood vessels were noted. In deeper tissue, there were numerous striated muscles and mucous acini with striated and excretory ducts. All features were suggestive of the normal architecture of the labial mucosa (Figure 6a, 6b).

## Discussion

Congenital double lip is an uncommon clinical abnormality that is characterized by excessive areolar tissue of the labial mucosa which may involve the upper and lower lip [5,11]. Chidzonga and Mahomva in 2006 [5] also reported a case of lower double lip in a two-week-old female infant [5]. The condition arises in the second or third week of gestation due to the persistence of tissue between the pars glabrosa and pars villosa or hyperplasia of the glandular tissue, which gives it a characteristic "cupid's bow" appearance [2,11]. Most cases involve both sides of the lip, but a few unilateral cases have also been reported in the literature [4]. Due to the attachment of the upper labial frenum, some individuals with double upper lip appear with a central constriction [7]. This malformation may be present at birth but becomes prominent after the eruption of permanent teeth [4]. Chronic swelling of the lip such as hemangioma, lymphangioma, angioedema, and cheilitis glandularis are the differential diagnosis of double lip [7,12,13]. Double lip is commonly associated with Ascher syndrome, which is characterized by a triad of double lip, blepharochalasis, and nontoxic thyroid goitre. It was first described by Karl Wolfgang Ascher, an ophthalmologist, in 1920 in a case report, and its aetiology is still unknown [6,13]. There have also been a few reported occurrences of double lip in conjunction with cleft palate, cheilitis glandularis, hemangiomas, and bifid uvula [1].

In the presented case, the patient did not have any clinical signs and symptoms of blepharochalasis and nontoxic goitre; therefore, Ascher syndrome was ruled out. The majority of cases are associated with aesthetic concerns, which can affect the appearance of the lips, leading to lower self-esteem, and only a few instances show functional problems such as difficulty in speech and eating. Aesthetic concerns can have a substantial impact on a patient's psychosocial well-being. Healthcare practitioners must address the emotional and psychological aspects of the condition and offer treatment as needed. The double lip abnormality has been treated surgically using several approaches which include the removal of excessive mucosa and underlying tissue to resolve functional and aesthetic problems. The surgical removal of an excessive fold of tissue is a well-documented treatment approach for this condition [4]. The majority of case reports in the literature used the elliptical incision technique which shows a high success rate [1-9,11,12]. Because of the reported success rate, an elliptical incision was used in this case; nevertheless, Guerrero-Santos and Altamirano also described that W-plasty can be used to yield similar results [11]. Peterson recommended electrosurgical excision and triangular excision as successful techniques for the treatment of double lip [9]. Troeltzsch et al. suggested a new technique with a mucosa-sparing, transvestibular approach to prevent lip shortening and tissue loss [12]. The 3-0 silk sutures were chosen because it has the highest resistance to tension and can withstand enough mechanical stress from muscle pull [14]. The microscopic examination of the aforementioned case revealed striated muscle fibres and mucous acini with striated and excretory ducts. Orbicularis oris muscle fibres may obstruct healing by mobilising the lip, resulting in unfavourable outcomes [4,9]. Most of the cases in the literature suggest that the only treatment modality to correct double lip is surgical removal [1-9,11]. The patient was satisfied with the postoperative outcome, and the healing process went without difficulty. A year after the procedure, the upper lip still looks healthy.

Since Cohen and Jago (1976) argued for the development of sociodental indicators, researchers have worked to create tools to quantify OHRQoL [15]. The subjective evaluation of OHRQoL considers an individual's comfort when eating, sleeping, and social interactions, as well as their self-esteem and oral health satisfaction. Oral health issues, social and contextual variables, and the rest of the body all combine to create this outcome [15]. The use of OHRQoL as an evaluation outcome measure is consistent with patient-centered care. It allows oral healthcare practitioners, in conjunction with other clinical examinations, to evaluate the efficacy of treatment protocols from the patients' viewpoints [10,15]. In the present case, OHRQoL has been measured before and after the treatment with the use of a questionnaire. In the present case, the overall satisfaction of the patient was excellent after the treatment. After the therapy, his concern about his appearance was allayed since he felt more confident, which kept him out of socially awkward situations. The current approach could successfully treat deformity without intraoperative or postoperative problems. There was no recurrence after 12 months; however, long-term follow-ups are required. Taking everything into consideration, the double lip is a rarely encountered abnormality that should be correctly identified and surgically treated. Although there are various additional conservative treatments, such as electrocautery and lasers, to treat double lip, they result in delayed healing.

## Conclusions

When excessive mucosal lip tissue interferes with mastication or phonetics or leads to the development of lip sucking or biting, treatment of double upper lip is indicated. A detailed aesthetic lip analysis before any surgical intervention is needed. A psychological evaluation of the person might be advisable. Prior to surgery, patient counselling is crucial for determining what the patient hopes to achieve from the procedure and for emphasising the need for appropriate follow-up. The importance of treatment is also considerable due to the patient's aesthetic concerns and psychological satisfaction.
